# Supplementary material for: Complexome profiling of the Chlamydomonas psb28 mutant reveals TEF5 as an early PSII assembly factor
Source: Plant Cell. 2025 Mar 17;37(6):koaf055. doi: 10.1093/plcell/koaf055 (PMC12164590; doi:10.1093/plcell/koaf055)
Supplement: koaf055_Supplementary_Data [file koaf055_supplementary_data.zip › psb28_interactive.zip!/plots/intensity/qualityPlots/peptides/_Cre12.g549350.t1.2.html]

### Cre12.g549350.t1.2

Trivial name:   
SeqCoverage [median]: 29.60  
Mass: 29.52 kDa  
  
 

| Sequence | MissedCleavage | Mass [Da] | wtIntensity | muIntensity | Score |
| --- | --- | --- | --- | --- | --- |
| AGGDAPAASTEAADAAVDAANAAR | 0 | 2112.97 | 33507.90 | 102668.19 | 168.84 |
| ALALAGAK | 0 | 713.44 | 2223.89 | 24287.53 | 122.15 |
| ELYGMAESALAK | 0 | 1281.63 | 0.00 | 2780.01 | 133.23 |
| SAAVGGELK | 0 | 830.45 | 28462.93 | 53843.94 | 147.36 |
| SAAVGGELKSSAAAAYEASSSSSTTR | 1 | 2445.16 | 0.00 | 910.21 | 47.38 |
| SSAAAAYEASSSSSTTR | 0 | 1632.72 | 11495.72 | 19518.29 | 287.76 |
| VKELYGMAESALAK | 1 | 1508.79 | 20945.97 | 47161.04 | 115.68 |
| YSAETNK | 0 | 811.37 | 8179.18 | 10974.30 | 145.04 |
|  |  |  | 104815.6 | 262143.5 |  |
